# Comparison of Two Cryogenic Radiometers at NIST

**DOI:** 10.6028/jres.106.029

**Published:** 2001-08-01

**Authors:** Jeanne M. Houston, David J. Livigni

**Affiliations:** National Institute of Standards and Technology, Gaithersburg, MD 20899-8442; National Institute of Standards and Technology, Boulder, CO 80303

**Keywords:** cryogenic radiometer, electrical substitution radiometry, silicon photodiode, trap detector

## Abstract

Two cryogenic radiometers from NIST, one from the Optical Technology Division and the other from the Optoelectronics Division, were compared at three visible laser wavelengths. For this comparison, each radiometer calibrated two photodiode trap detectors for spectral responsivity. The calibration values for the two trap detectors agreed within the expanded (*k* = 2) uncertainties. This paper describes the measurement and results of this comparison.

## 1. Introduction

At NIST, optical power determinations are based on the principle of electrical substitution radiometry, but also involve measurements of window transmission and characterization of instrument electrical-to-optical equivalence. Two cryogenic radiometers operating at NIST facilities in Gaithersburg, Maryland, and Boulder, Colorado, provide measurements of optical power using this technique. The High Accuracy Cryogenic Radiometer (HACR) was constructed in the Optical Technology Division [1]. The HACR is an absolute detector that is the basis for the optical scales of detector spectral response, photometry, radiance, and irradiance. The second device, the Laser-Optimized Cryogenic Radiometer (LOCR), is located in the Optoelectronics Division in Boulder [2]. The LOCR is one of several electrically calibrated, primary reference standards used by the Optoelectronics Division for laser power and energy measurements. Comparing a calibration based upon both the LOCR and HACR reference standards enables us to determine the level of consistency between these two measurement standards and to develop increased confidence in the quality of the complementary measurement services provided at each of the locations. The HACR is an instrument based on the prototype developed at the National Physical Laboratory (NPL) [3] in the 1980s. The parts were assembled by NIST personnel at NPL and in Gaithersburg. The LOCR is a custom design, built to NIST specifications by Cambridge Research & Instrumentation, Inc^1^. The LOCR is similar in design and operation to an instrument developed by the manufacturer in the early 1990s [4].

These cryogenic radiometers are based on electrical substitution radiometry (ESR) and operate at liquid helium temperatures. They are optimized for the measurement of laser power. Electrical substitution radiometry determines the optical watt from the electrical watt by optically heating an absorbing cavity, and substituting the optical heating with electrical heating. The electrical power determined by straightforward electrical measurements is then equated to the optical power. To minimize the nonequivalence between the electrical and optical heating, the design incorporates features to reduce the thermal gradients in the optical cavity and to minimize conduction and convection losses. Operation at liquid helium temperatures improves the power sensitivity by lowering the heat capacity of the optical cavity and by reducing the thermal noise. In addition the use of superconducting wires eliminates error-producing losses in the electrical circuit. A full description of the uncertainties related to these factors can be found in Refs. [1] and [2].

A direct comparison of HACR and LOCR, with one radiometer next to the other measuring the same source of optical radiation, was not possible due to instrument size and location. The next best solution was to compare indirectly using calibrated artifacts, as was done in recent international comparisons of cryogenic radiometers [5, 6]. For this comparison, the artifacts were two silicon photodiode trap detectors. A trap detector is a configuration of several silicon photodiodes geometrically arranged to keep reflecting the incoming light onto their active areas to improve the light collection efficiency to near 100 %. Both HACR and LOCR calibrated these trap detectors and their values were compared.

## 2. The Instruments and Optical Setup

There are relevant similarities and differences in the HACR and LOCR instrumentation and measurement protocols. Details can be found in Refs. [1, 2, 5, and 7]. For this comparison the relevant similarities are the shape of the receiving cavities and the dynamic range of the instruments. Additionally, each ESR uses translation stages to position the transfer devices into the optical beam, allowing for multiple instrument calibrations in a cycle. The major differences include the sizes of the receiving cavities and their resulting time constants, the electrical and optical substitution methods, the direction of the optical axes (vertical or horizontal), and the type of temperature measurement system.

The input geometry of HACR’s optical axis limits the type of transfer detectors that may be used for a comparison. Due to HACR’s vertical input, there is a clearance of 10 cm for transfer devices. Additionally, the vertical beam path requires a mirror to steer a beam originally parallel to the table surface into the receiving cavity. This 45° steering mirror is subject to dust contamination. The vertical cavity also requires that the transfer device be placed some distance in front of the HACR’s cavity, resulting in a slightly different intensity profile. To ensure total collection of the optical radiation by both instruments, which is crucial to the calibration transfer, HACR requires a beam diameter that underfills the transfer detector’s active area and HACR’s limiting apertures.

LOCR is designed with the cavity accepting a horizontal optical path, eliminating the steering mirror and the limitation on the transfer detector size. Additionally, LOCR is on a translation stage so the trap detector can move into the laser beam in the same optical plane, allowing both devices to be exposed to the same beam profile.

Another difference between the two systems is the temperature measurement electronics. While LOCR uses ac bridge electronics located directly under the cryostat, HACR uses a dc measurement of the temperature. The ac measurement and the close location of the electronics produces a lower noise temperature measurement.

The optical systems of HACR and LOCR are similar. Intensity-stabilized, spatially filtered, single wavelength lasers are used as sources. For both sets of measurements, the laser lines used were the argon ion laser lines at 488 nm and 514 nm, and the helium-neon laser line at 633 nm. The optical systems produce beams with approximately Gaussian intensity profiles.

**Fig. 1:**
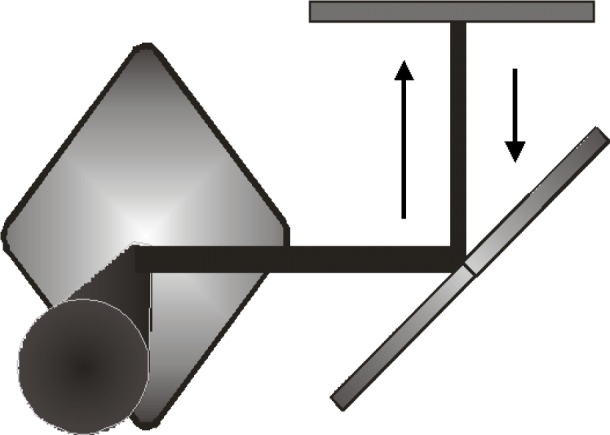
A three Si photodiode reflectance trap detector. Shown is its optical path, which includes the five surface reflectances.

Both NIST Boulder and NIST Gaithersburg supplied a trap detector for the comparison. Each trap was a three photodiode reflectance configuration shown in Fig. 1 [8, 9]; one that was commercially built and another built in the Optical Technology Division in Gaithersburg. The devices were selected to be the best that were available at the time of the measurements and not built or designed specifically for this intercomparison.

## 3. The Measurements

HACR and LOCR measured the two trap detectors, labeled TS02 and NIST 6 using the optical setups, protocols, and uncertainties described in their respective papers [1, 2]. The two ESR’s used different electrical and optical substitution methods. With the HACR, the optical watt is determined from the electrical watt by optically heating an absorbing cavity, then creating the same temperature change with electrical heating. Three heating cycles are performed to transfer the calibration from the cryogenic radiometer to the trap detector, one optical and two electrical. In the two electrical heating cycles, the receiving cavity is heated to close to the temperature achieved by optical heating. The optical power is calculated using a linear interpolation from the two electrical power measurements. In a direct calibration transfer, the trap detector measures the same optical power as HACR by moving it into the beam just preceding the radiometer window [10]. The optical powers measured and determined by HACR are used to calibrate the trap detector’s spectral responsivity in the units of amperes/watt. The calibration of the two traps included values taken over a period of 5 days to 8 days for each wavelength. To account for the uncertainties in locating the center of the detectors’ active areas, each set of measurements included 3 to 5 different trap alignments. Approximately 100 measurements at each wavelength are included in the data analysis.

With the LOCR, the absorbing cavity and the cavity heat sink are maintained at constant temperatures by electrical control systems. The absorbing cavity is maintained at a temperature of approximately 5.4 K. The electrical substitution is performed by measuring the drop in the amount of electrical heating power that is required to maintain the cavity temperature when the optical power is applied. Since the cavity temperature ideally never changes, and the electrical nonequivalence and resulting thermal gradients are negligible, the LOCR’s response time is determined primarily by the response time of the electronic control system, and is not limited by the heating time constant of the cavity. Therefore, the LOCR can measure the absolute optical power relatively quickly.

The LOCR’s optical power measurement is transferred by physically substituting the trap detectors into the beam path, the same plane in which the LOCR’s cavity was located. The trap detector’s response to the beam is then measured. By using the optical power determined from the LOCR measurements, the spectral responsivity of the traps is calculated at that specific wavelength [7]. A total of 4 to 10 measurements were performed for each trap detector at each wavelength.

The trap detector TS02 was originally sent to Boulder from Gaithersburg for comparison. In Boulder, the trap detector TS02 and the Boulder-supplied NIST 6 were calibrated at 3 wavelengths. The two trap detectors were then sent to Gaithersburg for calibration. For these measurements, the relevant laser beam specifications at both Boulder and Gaithersburg, for all three wavelengths, ranged in power from 0.25 mW to 1 mW. The beams for all three wavelengths had a 1/e^2^ intensity diameter of about 2 mm at the entrance to the traps.

While the methods of measuring optical power are similar for both LOCR and HACR, there is a critical difference in the procedures used to align the trap detectors. The trap detector alignment for LOCR was a visual alignment to the center of the entrance apertures, with the laser beam within a few mrads of normal incidence relative to the trap’s entrance aperture. For the trap TS02, the laser was aligned to the aperture cover’s cross hairs with an uncertainty of less than 0.5 mm. NIST 6 has no aperture cap and a scale locates the center to an uncertainty of less than 1 mm.

For HACR measurements, there is a program for automated alignment. First, the trap detector is angularly aligned so that the residual laser beam is retroreflected. Then for each axis, *x* and *y*, the trap detector is scanned in 0.2 mm steps and the center is located halfway between the 80 % points of the peak. The uncertainty of this alignment is less than 0.2 mm. It is not uncommon that the center of the active area, as determined by this protocol, is not the physical center of the entrance aperture. If a detector is not measured with the optical beam in the same location within its aperture, the uncertainty of the comparison is affected by the spatial uniformity of the detector. Also, the potential difference in the laser’s incident angle in the HACR and LOCR calibrations resulted in a slightly different measured spatial uniformity for the traps. When rotated over an angular range of ±0.04 radians around the center of the active area, the two traps showed a variation of ±0.02 % in response. The combination of the spatial nonuniformity and angular alignment probably contributed to the difference in the measured calibration factors.

## 4. Results

**Fig. 2:**
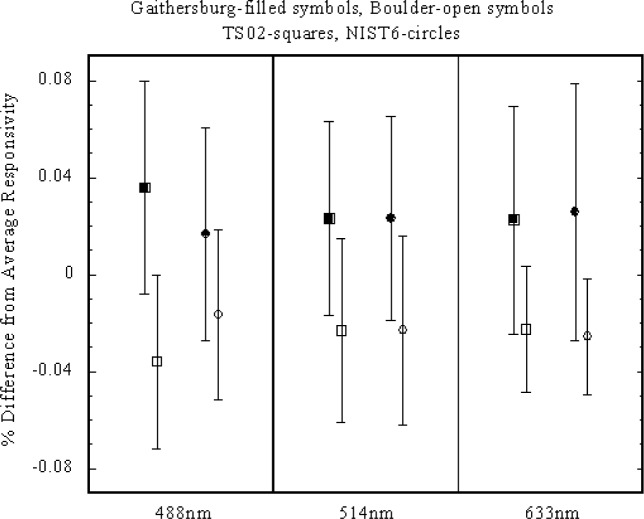
Comparison of the measured absolute responsivities [A/W] for trap detectors TS02 and NIST 6. The values are normalized to their average and shown with the expanded (*k* = 2) uncertainties.

The results of the measurements are listed in Table 1. As shown in the table there is a difference in the calibration values of 0.05 % at 633 nm to 0.07 % at 488 nm. Figure 2 shows the Boulder and Gaithersburg values normalized to their average and plotted with their respective *k* = 2 uncertainties. This graph shows that the measurements are within their respective uncertainties.

These uncertainties do not reflect any effects of trap detector spatial nonuniformities or incident angle, but only the uncertainties in calibrating a trap detector from HACR or LOCR (Tables 2 and 3). The trap detectors were measured for spatial uniformity both at Boulder and at Gaithersburg.

Before the start of any calibrations at Boulder, the traps were blown clean using an inert dusting gas. The devices were measured for spatial uniformity before the start and after the conclusion of the calibrations. The Boulder spatial uniformity measurements were completed using the Spatial Uniformity Scanning System [11] with a 2 mm diameter beam, produced by a 635 nm, fiber-coupled laser diode. The beam was raster-scanned in 0.2 mm steps; to reduce the noise, 9 scans of TS02 and 16 scans of NIST 6 were averaged. The traps were aligned as in the LOCR measurements, with the incoming beam at near normal incidence to the entrance aperture, and the center of the beam aligned to the nominal center of the aperture. The trap’s response had a peak-to-peak variation of 0.025 % for TS02, and 0.035 % for NIST 6, within 1 mm of the nominal center. The scans revealed no significant problems in the spatial uniformity.

At Gaithersburg, the traps were measured for spatial uniformity at the beginning of the calibrations. After their arrival in Gaithersburg, the traps were not blown clean to maintain the detectors in the condition that they were measured in Boulder. Cleaning a detector can change the surfaces and therefore alter the resulting device responsivity being measured. The Gaithersburg spatial uniformity measurements were completed in the Laser Comparator Facility [12] using a 2 mm diameter, 633 nm HeNe laser. The beam was raster-scanned across a 10 mm by 10 mm area in steps of 0.5 mm. Each point in the scan is an average of five samples. The traps were aligned using the same procedure for the HACR measurements, with the center of the active area being halfway between the points that were 80 % of the peak. The residual beam from the traps was retroreflected.

The results of the spatial uniformity mappings at Gaithersburg show that both TS02 and NIST 6 suffered contamination during transport. TS02 had the largest uniformity problem, with a pit, caused by dust, whose responsivity was approximately 0.78 % less than the peak responsivity in the central portion of the active area. Other than this pit, the uniformity of the detector was better than 0.03 % over the central active area. NIST 6 did not have any obvious large dust particles in the center of its active area, but its spatial nonuniformity over that critical region was on the order of 0.05 %. The existence of the dust for both detectors was visually confirmed.

The dust spot on the center of TS02’s active area caused problems in the Gaithersburg calibrations. Measurement repeatability suffered due to the laser beam hitting and scattering from the particle. After reviewing the Gaithersburg uniformity plots, the alignment method for TS02 was changed to move the aligned laser beam by 1 mm below the located center to avoid the dust and remain in the center of the uniform active area. The standard deviations improved after this change was implemented.

Additional elements affecting the uncertainties include the laser stabilities. In the Gaithersburg measurements, the noise on the laser stability at 633 nm was ±0.01 % with a slow drift and ±0.02 % for the argon laser lines. For HACR, the short-term noise is seen by the trap detectors but averaged out by the radiometer’s long time constant. The Boulder measurements had smaller standard deviations partially because the calibrations were performed in a single day. Also, the temperature, pressure, humidity, and resulting air index were significantly different at the two laboratories.

## 5. Summary

The comparison of LOCR and HACR, the two independently developed cryogenic radiometers at NIST, showed agreement within their measurement uncertainties, although the difference of 0.04 % to 0.07 % between their calibration values was greater than desired. If one considers the trap detectors’ spatial uniformities, the difference in the alignment procedures, and the different laboratory environments, the differences in the calibration values are not unreasonable.

Experience with this comparison suggests improvements that could be made in a future comparison. One improvement is to develop a more detailed measurement protocol that addresses the difficulties in the comparison. Additionally, we are designing and building new trap detectors that are more spatially uniform and would incorporate an alignment technique that is viable for both radiometers. Evaluation of any uncertainties resulting from the different laboratory environments should also be considered.

Presently there are developments in trap detector designs at both Boulder and Gaithersburg that could lead to better comparison detectors. These devices could be designed with alignment jigs that define the point of alignment and would reduce uncertainties due to detector spatial uniformity. Also in Gaithersburg, a second generation cryogenic radiometer called HACR 2 is being built. This radiometer will incorporate AC electronics for the temperature measurement to lower the system noise and uncertainties. Additionally, HACR 2 is designed so that the receiving cavity is parallel to the table surface. This improvement eliminates the 45° steering mirror from the optical path and removes the physical limitations placed on the transfer devices presently required by HACR.

With the improvements mentioned and the new instruments, it is expected that future comparisons between LOCR and HACR 2 would show a great improvement. Once the new traps and HACR 2 are operational, a comparison between the two laboratories is anticipated.

## Figures and Tables

**Table 1:** Comparison of the HACR and LOCR calibration values for two trap detectors. The percent differences are calculated using: ((Gaithersburg or Boulder−value mean)/mean) × 100

Detector	488 nm		514 nm		633 nm	
	Gaithersburg	Boulder	Gaithersburg	Boulder	Gaithersburg	Boulder
TS02						
Absolute Response (A/W)	0.3903	0.39002	0.41221	0.41202	0.5085	0.50827
Expanded % Uncertainty (k = 2)	0.044	0.036	0.04	0.038	0.047	0.026
% Difference from average	0.036	−0.036	0.023	−0.023	0.023	−0.023
NIST 6						
Absolute Response (A/W)	0.38969	0.38956	0.41172	0.41153	0.5080	0.50774
Expanded % Uncertainty (k = 2)	0.044	0.035	0.042	0.039	0.053	0.024
% Difference from average	0.017	−0.017	0.023	−0.023	0.027	−0.027

**Table 2:** Components in the combined relative standard uncertainty of HACR optical power measurements. A full discussion of the corrections and uncertainties can be found in Ref. [1]

Type	Wavelength dependent uncertainty (%)			Correction	Uncertainty (%)
Type A (*N* = 94 to 180)	488 nm	514 nm	633 nm		
TS02	0.008	0.008	0.013		
NIST 6	0.008	0.008	0.018		
Type B, Combined					
Window transmittance	0.008			0.99976	0.008
		0.005			0.005
			0.005		0.005
Scattered Optical Power	0.013			+72 nW	0.013
		0.010		+66 nW	0.010
			0.010	+47 nW	0.010
Cavity Absorptance				0.99998	0.002
Nonequivalance				1.00000	
Temperature Gradiants					0.004
Heater Power					
*V*_H_, *V*_R_					0.003
*R*					0.0003
Amplifier gain					0.010
Voltage measurement					0.003

Combined uncertainties for:					
TS02	0.022	0.020	0.023		
NIST 6	0.022	0.021	0.026		

**Table 3:** Components in the combined relative standard uncertainty of LOCR optical power measurements. A discussion of the corrections and uncertainties can be found in Ref. [2] and [11]

Type	Wavelength dependent uncertainty (%)			Typical value of correction	Component of uncertainty (%)
Type A (*N* = 4, 9 or 10)	488 nm	514 nm	633 nm		
TS02	0.0009	0.0005	0.0003		
NIST 6	0.0026	0.0025	0.0004		
Type B, Combined					
Window transmittance	0.0063			0.999787	0.0063
		0.0038		0.999820	0.0038
			0.0053	0.999950	0.0053
Cavity Absorptance	0.0050			0.999821	0.0050
		0.0050		0.999871	0.0050
			0.0002	0.999920	0.0002
Aperture Transmittance					
TS02	0.0134	0.0119	0.0063	0.999990	
NIST 6	0.0068	0.0052	0.0044	1.000014	
LOCR Alignment	0.0059	0.0118	0.0074		
Nonequivalance					0.0002
LOCR Electronics @ 0.25 mW				0.999999	0.0113
LOCR Electronics @ 1 mW				0.999999	0.0028
Amplifier gain					0.0058
Voltage measurement					0.0006

Combined uncertainties for:					
TS02	0.018	0.019	0.013		
NIST 6	0.018	0.019	0.012		

## Footnotes

1 Certain commercial equipment, instruments, or materials are identified in this paper to foster understanding. Such identification does not imply recommendation or endorsement by the National Institute of Standards and Technology, nor does it imply that the materials or equipment identified are necessarily the best available for the purpose.
